# ‘No one prepared me to go home’: Cerebrovascular accident survivors’ experiences of community reintegration in a peri-urban context

**DOI:** 10.4102/phcfm.v11i1.1806

**Published:** 2019-04-24

**Authors:** Pragashnie Govender, Deshini Naidoo, Kiara Bricknell, Zainab Ayob, Holly Message, Sibongiseni Njoko

**Affiliations:** 1 School of Health Sciences, College of Health Sciences, University of KwaZulu-Natal, Durban, South Africa

**Keywords:** community reintegration, CVA survivors, occupational therapy, rehabilitation, service delivery

## Abstract

**Background:**

The South African health system has policies and strategies to ensure effective rehabilitation and reintegration of individuals who have survived a cerebrovascular accident into their respective communities. However, implementation of such guidelines remains an issue.

**Aim:**

This study sought to explore cerebrovascular accident (CVA) survivors’ experiences of community integration.

**Setting:**

The study was located in a peri-urban community within the KwaZulu-Natal Province, South Africa.

**Methods:**

An explorative qualitative study with eight purposively selected CVA survivors was conducted via semi-structured individual interviews. Data were audio-recorded and manually transcribed prior to thematic analysis. Trustworthiness of the study was maintained by strategies such as analyst triangulation, an audit trail and use of thick descriptions. Ethical principles of autonomy, informed consent, confidentiality and privacy were also maintained in the study.

**Results:**

Six themes emerged that highlighted (1) loss of autonomy and roles, (2) barriers to community reintegration, (3) social isolation of participants, (4) finding internal strength, (5) enablers of community reintegration including the positive influence of support and the benefits derived from rehabilitation and (6) recommendations for rehabilitation.

**Conclusion:**

The study revealed both positive and negative influences that impact CVA survivors’ ability to effectively reintegrate into their respective communities following a CVA. Recommendations include the need for education and awareness around access to rehabilitation services for CVA survivors, advice on how to improve CVA survivors’ ability to mobilise in the community and make environmental adaption to facilitate universal access, provision of home programmes and caregiver training for continuity of care and for inclusion of home-based rehabilitation into current models of care.

## Introduction

Cerebrovascular accidents (CVA) are one of the leading causes of death and disability worldwide, with the incidence rate in South Africa (SA) being second only to HIV and AIDS.^1,2^ Several authors have highlighted that CVA survivors experience multiple impairments that negatively influence their ability to participate in their premorbid daily activities.^3,4,5,6,7,8^ The South African health system has policies and strategies that guide rehabilitation and community reintegration, an example of which is the *Framework and Strategy for Disability and Rehabilitation*^9^; however, implementation of such guidelines remains an issue.^10,11,12,13,14^ Despite the focus of the Department of Health on reengineered primary health care (PHC), CVA survivors still have difficulty accessing rehabilitation services.^11,12,13,14^

Comprehensive rehabilitation spans inpatient rehabilitation at a regional or district hospital to services offered at PHC centres and in the community. The integration of rehabilitation services and the PHC approach (community-based rehabilitation, CBR) has been described as a positive service geared to deliver a rehabilitation service and empower communities to take ownership of their health and wellness despite the lack of available resources.^15^ For CBR services to be successful, a clear understanding of the expectations of the clients is required, as well as rehabilitation professionals who can identify community resources and enable clients to be more autonomous.^12,13,14^ Dijker^16^ defined *community reintegration* as acquiring age-, gender- and culturally appropriate roles and activities in one’s natural community setting. Community reintegration remains a key goal for rehabilitation professionals as it focuses on the CVA survivor’s ability to function, participate in premorbid life roles (which form part of the survivor’s identity) and contribute to the community.^4,5,13,15,17^ Numerous authors have highlighted the poor reintegration among South Africans, who have had a CVA or a spinal cord injury, into their respective communities.^4,8,17,18,19^ Gretschel et al.^4^ found that adults with a disability face challenges with participating in work or meaningful activities, community mobility and recreation. Similarly, Carwood et al.^18^ and Pang et al.^20^ highlighted the difficulties experienced with participation in social, leisure, work and community activities. Rouillard et al.,^7^ Wassermann et al.^8^ and Cunningham and Rhoda^3^ concurred that having a CVA negatively impacts on the CVA survivor’s lifestyle, with participants in their studies experiencing difficulty resuming their previous family roles and having difficulty engaging with work and being able to negotiate around their community. In a review of the available literature, only one study highlighted the experiences of CVA survivors 3 months post-discharge in a rural area in KwaZulu-Natal (KZN). The aim of this study was to explore community reintegration experiences of CVA survivors in a peri-urban area in KZN and the factors that facilitate or hinder this reintegration.

## Research methods and design

### Study design

This study followed an explorative qualitative design^21^ with the use of semi-structured interviews to gain greater insight into the experiences of CVA survivors in their process of community reintegration.

### Study location

The study was located in the peri-urban community of KwaDabeka in KZN. Peri-urban townships are described as the area in between rural and urban areas. Because of the segregation caused by the spatial planning strategy of apartheid, townships such as KwaDabeka arose as solely black residential zones during this era. KwaDabeka’s close proximity to two major intersections makes this community one of the well-located previously disadvantaged areas in eThekwini for those seeking employment. Typically, the households in this community have a low level of skill, with 75% of the population not having completed secondary education. This often results in the average household income being an estimated R15 917.00 per annum (approximately R1325.00 per month), with an average of 3.5 people per household.^9^ This income is considered below the national poverty line. There is one community health care centre (CHC) in KwaDabeka, with eight satellite clinics, which services a population of 232 877. There is a multidisciplinary team at the CHC that includes nurses, occupational therapists, physiotherapists, dental therapists and a dietician as well as community health care workers (CHWs). The main referral hospitals include R.K. Khan, a regional and district hospital with a multidisciplinary team, and St Mary’s, a district-level hospital.

### Study population and sampling strategy

The target population included persons who had suffered a CVA who were currently residing in the KwaDabeka community. Non-probability purposive sampling^21^ was used to recruit eight participants via CHWs functioning within the CHC. As there were no available records to determine the number of potential participants, the size of the target population could not be determined. Participants had to have suffered a CVA, be over the age of 18 years, currently reside within the community and have the ability to communicate in either isiZulu or English. Those CVA survivors who were unable to communicate were excluded from the study. The participants selected were CVA survivors who had volunteered to participate, after explanation of the aims of the study, during door-to-door recruitment or based on recommendations from CHWs. The participants who volunteered met the inclusion criteria and were able to offer their perspective on the research questions.

### Data collection and data analysis

A pilot study was conducted with one CVA survivor, with minor changes to the interview schedule and inclusion of more open questions to ensure opportunity for a deeper conversation led by the CVA survivors on how they experienced community reintegration following their CVA. Data were collected using a biographical survey and semi-structured interviews. Of the eight interviews, seven were conducted in isiZulu and one in English. Interviews occurred within the CVA survivors’ homes or at the CHC. Interviews spanned 45 min to an hour and were audio-recorded. The interviews were translated and transcribed by two of the authors and analysed thematically^22^ by all authors who identified codes, categories and themes. Analysis occurred prior to the ensuing interview to ensure that sampling ceased once redundancy was reached.

### Trustworthiness and rigour

In order to maintain trustworthiness,^15^ an audit trail was maintained and verbatim quotes were used to ensure the veracity of the data. Analysis by four of the authors followed by discussion and refinement and consensus allowed for analyst triangulation. Sufficient information about the context of the study is also described to allow for transferability.

### Ethical considerations

Ethical approval was obtained from the Humanities and Social Sciences Ethics Committee and the KwaZulu-Natal Provincial Health Research and Knowledge Management Directorate that processes health-related ethical applications for the province. Gatekeeper permission was gained from the eThekwini District and the KwaDabeka Community Health Clinic. Participants were informed of the procedure of the study, signed informed consent was solicited prior to commencement of the interviews and measures for counselling were in place as a caution if participants became distressed during the interview. Privacy and confidentiality were maintained through ensuring that each participant had a pseudonym allocated with no identifying data to match the participant to the transcript.

## Findings

The experiences of eight CVA survivors were explored. Five male and three females, ranging from 38 to 79 years, were accessed, of which seven had experienced their CVA between 2016 and 2017. Table 1 outlines specific demographics of the sample.

**TABLE 1:** Demographics of participants (*n* = 8).

Variable	Buhle	Sbo	Joyce	Musa	Phindile	Thandi	Ziyanda	Xolani
Age	63	38	56	51	76	69	55	41
Gender	Female	Male	Female	Male	Female	Female	Female	Male
Race	Black	Black	Black	Black	Black	Black	Black	Black
Educational level	Grade 8	Grade 12	Grade 11	Tertiary	Grade 10	Tertiary	Grade 7	Grade 12
Prior living arrangements	Family	Mother and wife	Family	Children	Family	Children	Family	Girlfriend
Current living arrangements	Family members	Wife	Family	Children	Family	Children	Family	Girlfriend
Date of stroke	2016	2016	2014	2016	2017	2016	2016	2016
Side of hemiplegia	Left	Left	Left	Right	Left	Right	Left	Right
Post-CVA hospital admission	2 weeks	None	None	6 days	7 days	5 days	5 months	2 weeks
In-hospital rehabilitation	None	None	None	Yes	None	Yes	Yes	Yes
Referral to community clinic	No	No	Yes	No	No	No	No	No
Community rehabilitation	None	Yes (2017)	Yes (2015–2017)	Yes (2017)	Yes (2017)	Yes (2016–2017)	Yes (2017)	Yes (2017)
Employed prior to stroke	Retired	Yes	No	Yes	Retired	Retired	Yes	Yes
Current work status	Retired	No	No	Yes	Retired	Retired	No	Yes
In receipt of disability grant	No	Yes	Yes	No	No	No	Yes	Yes

Six themes emerged that are described in the following section, with verbatim quotes to highlight the participants’ voices. Pseudonyms have been used.

### Theme 1 – Loss of autonomy and roles experienced: ‘Life is different’

Participants reported feeling a sense of dependency following their CVA. They experienced a loss of autonomy, as they were constantly seeking help from others and were reliant on family members to assist them to perform the basic activities of daily living (ADL). They could no longer go alone into the community. Their choice of how they participated in community activities was limited, because of their dependence on other people:

‘I am always asking for people to help, and I don’t like that.’ (Buhle, age 63, female, grade 8, retired)

‘I used to hate going to the toilet and going to bathe because it is not a nice feeling to have them bathe you and help you in the toilet …’ (Phindile, age 76, female, grade 10, retired)

‘I was all dressed up to go to church, and I didn’t have someone to take me.’ (Thandi, age 69, female, tertiary level education, retired)

Four participants worked prior to their CVA and only two were able to return to work, with those able to return to work experiencing a reduced number of tasks within their previous job functions. All experienced some loss in social roles, which included restricted participation in their premorbid community, family or church functions as well as environmental factors that restricted participation, such as stairs. Moreover, the participants voiced not being able to care for their family, as many roles were now reversed or taken over by family members:

‘Before the stroke, [as my work] I was part of a team that used to go to the community and help people in need … But now I [can] only do paperwork and give out medication.’ (Xolani, age 41, male, grade 12, employed)

‘… even if I do go to church, I sit in the benches and I can’t sing and dance as before. It makes me not want to go to church because people start feeling sorry for me because I am not as active as before.’ (Ziyanda, age 55, female, grade 7, employed)

‘There is a big difference between now and before, especially because I am a married woman – I can’t take care of my husband; I need to be cleaning, cooking and washing his clothes but I can’t do all that because of my condition.’ (Buhle, age 63, female, grade 8, retired)

‘That’s why I don’t go most of the time to functions. Because I think about the conditions there. I think about the steps, I think about everything.’ (Musa, age 51, male, tertiary level education, employed)

### Theme 2 – Barriers to community reintegration

Fears of falling, of being mugged or attacked, of going to places alone and of going to the hospital were experienced as environmental or contextual factors or barriers that limited community reintegration. Some participants also experienced stigma in the community, whilst others thought they were cursed:

‘I’m scared of falling, because I once fell trying to get down [the steps].’ (Sbo, age 38, male, grade 12, employed)

‘I can’t go alone, because I am scared. Say I’m going to the bank; it is not safe.’ (Thandi, age 69, female, tertiary level education, retired)

‘Some people believe it is bad luck to be near someone like me, because they believe I am cursed.’ (Sbo, age 38, male, grade 12, employed)

The participants reported that there were factors in the environment that limited participation such as steps and steep hills and roads, either in their home or the larger community, as well as the lack of affordable transport. All of these factors limited community reintegration and restricted their access. Additionally, the residual physical deficits post-CVA such as fatigue and weakness were factors that were limiting their participation in activities:

‘The community is limiting for me as a whole because of the many steps and hilly roads.’ (Ziyanda, age 55, female, grade 7, employed)

‘It [life] was hard because I can’t work with one hand.’ (Joyce, age 56, female, grade 11, unemployed)

‘Not having a car was very difficult … We have to take taxis; it was difficult and it was costing us because in the taxis we had to pay extra [the participants have to pay for the wheelchair and their place in the taxi].’ (Buhle, age 63, female, grade 8, retired)

Participants highlighted that they were not adequately prepared for return to home. Neither the CVA survivors nor their caregivers were provided with sufficient information about their condition, coping strategies or advice on environmental adaptations that would help with reintegration. Participants highlighted various obstacles to rehabilitation such as lack of access to rehabilitation in-hospital and in the community as well as a lack of referral following discharge:

‘No one [in the hospital] prepared me to go home. When I was at home it was even harder for me because I was very weak at that time and none knew how to take care of me.’ (Phindile, age 76, female, grade 10, retired)

‘I am still trying to settle in the community, because my family and I don’t fully understand my condition.’ (Sbo, age 38, male, grade 12, employed)

‘When I was in hospital I did not get any rehabilitation, but they promised me that the physiotherapist would come; but no physio came to me and I was later discharged.’ (Phindile, age 76, female, grade 10, retired)

‘I wish I had heard earlier about [rehabilitation at the clinic] because I have already seen some improvements in my life [after receiving therapy].’ (Ziyanda, age 55, female, grade 7, employed)

### Theme 3 – Social isolation: ‘I’m always alone’

Participants expressed that after their CVA they felt isolated. Many participants felt they were stuck in one place and that they were not able to leave their house and mobilise within the community:

‘I can’t go to different places like before; my life is stuck in one place.’ (Sbo, age 38, male, grade 12, employed)

‘I don’t usually leave the house.’ (Phindile, age 76, female, grade 10, retired)

‘I hated being bottled up, I hated not going to work, I like to be with people, to be with my friends. I hated being at home the whole day.’ (Xolani, age 41, male, grade 12, employed)

### Theme 4 – ‘I feel more like myself again’

Participants reported feeling positive emotions as a result of improvements because of therapy and the positive influence on their participation in daily activities. Emotions such as hope and joy assisted in their adjustment into the community, as they had a new appreciation for life:

‘I am happy because I feel like I am going back to my old self, and that to me is a big deal because it means that the exercises that I do at home and the clinic are working.’ (Xolani, age 41, male, grade 12, employed)

‘I look at life differently. At least I am alive. You can’t have everything you want and like. If it is meant to be, let it be. I have tried to cope with it.’ (Thandi, age 69, female, tertiary level education, retired)

‘But the point is we must always be positive because if we are not positive you won’t do a thing. Don’t feel sorry for yourself.’ (Musa, age 55, male, tertiary level education, employed)

‘The improvements make me proud of myself because I have been trying hard and pushing myself; I am happy.’ (Joyce, age 56, female, grade 11, unemployed)

Many participants expressed joy in resuming premorbid activities and roles. These activities included basic tasks such as eating, dressing, bathing, toileting, grooming and mobility as well as more complex tasks such as cooking and cleaning, shopping and returning to work:

‘I feel like I am my old self again because I can do things around the house [cooking and cleaning] that I wasn’t doing the time I was very sick. I feel like I am a mother again to my children.’ (Ziyanda, age 55, female, grade 7, employed)

‘Yesterday was the first time I went to church by myself, and it felt good.’ (Thandi, age 69, female, tertiary level education, retired)

‘It feels good to be able to wake up in the morning and go to work again; I never liked staying at home the whole day watching TV.’ (Xolani, age 41, male, grade 12, employed)

### Theme 5 – Enablers of community reintegration

Participants highlighted that they received support from their families, churches, neighbours and friends. The support they received from these individuals included well wishes, prayer, home visits and communion from the church. This social support motivated participants, as it displayed support from the community for the CVA survivors, which assisted in their coping with their circumstances. Those participants who were working also expressed that support was received from work, as their employer provided them with leave for rehabilitation and made adaptions to their work schedule and tasks to accommodate their return to work:

‘Seeing people [friends and neighbours] reaching out and coming to visit me at home has been not only good for me but for the family, because we needed everyone’s support … people came and showed us love.’ (Buhle, age 63, female, grade 8, retired)

‘[The support] helped me a lot; [my family] motivated me a lot – they were there for me when I had no hope of improving. They really motivated me.’ (Ziyanda, age 55, female, grade 7, employed)

‘At work they are good … if there is no improvement they will give me maybe another month or two.’ (Musa, age 55, male, tertiary level education, employed)

The participants appreciated receiving rehabilitation and acknowledged the benefits achieved because of participation in rehabilitation. Participants expressed that there were improvements in their day-to-day functioning because of their use of assistive devices and equipment, such as wheelchairs and environmental adaptions such as ramps. Improvements were also because of modified methods in carrying out daily life tasks, such as wearing loose clothing to make dressing and undressing easier. Insight into their condition was also observed, as participants were compliant to their medication intake and their use of the environment, such as steps, for exercising. Participants frequently expressed that the improvements in their ability from post-hospital to current functioning was because of rehabilitation provided by the physio and occupational therapist in the community:

‘Having a ramp built has been better than before because they don’t carry me like a child anymore; they push me with the wheelchair up the ramp.’ (Buhle, age 63, female, grade 8, retired)

‘When I am dressing it takes me a long time; that is why I now have decided to only wear loose clothing, because it is easier to put on.’ (Phindile, age 76, female, grade 10, retired)

‘I had a problem with wearing the bra, but now the OT [occupational therapist] showed me what I can do, and now I wear the bra.’ (Thandi, age 69, female, tertiary level education, retired)

‘I have been attending rehab here at the clinic and so far it has helped me a lot because I am far better than what I was before; I have seen a lot of improvement since starting it.’ (Xolani, age 41, male, grade 12, employed)

### Theme 6 – Recommendations for rehabilitation services

Participants expressed a desire for rehabilitation services within their home environment, as they were often unable to access the CHC. They also suggested that they would have benefited from home exercises and caregiver training to ensure carry-over of intervention from hospital to the community. Participants suggested that there should be referrals for rehabilitation from the hospital, as well as referrals to the CHC, given the distance of the referral hospital. They also expressed the need for earlier access to rehabilitation:

‘Right now, I don’t go [to the clinic and hospital] as frequent[ly] as before. I can’t go; it is far and there is no one to take me.’ (Thandi, age 69, female, tertiary level education, retired)

‘[The hospital] didn’t refer me [to rehabilitation in the community].’ (Thandi, age 69, female, tertiary level education, retired)

‘I want to continue doing exercises here at home; because I can’t go to the clinic, it is better if they would come and see me here at home.’ (Buhle, age 63, female, grade 8, retired)

‘If [the OT student] had come here [home] earlier, like in the beginning of my stroke, maybe I would be walking now, because she only saw me for [a] few weeks and I was able to cook for my family yesterday.’ (Buhle, age 63, female, grade 8, retired)

‘After coming back from the hospital, I didn’t know how to do most things; my family did not know anything [about a stroke and methods to assist with care].’ (Musa, age 55, male, tertiary level education, employed)

## Discussion

In this study, there were residual impairments that influenced CVA survivors’ ability to participate meaningfully in ADL. Activity limitations have been acknowledged as a barrier to participation.^3,4,6,7,8,20^ These activity limitations lead to difficulty in mobilising around the home and community, as well as the ability to engage in meaningful activities. This results in difficulty in resuming various roles and participation in premorbid activities. This lack of activity, loss of autonomy and social isolation that were experienced by the participants in this study can result in negative emotions such as hopelessness and helplessness. These factors inevitably impacted on the CVA survivors’ ability to regain independence in their roles at home, at work and in social participation. In turn, these role losses led to role reversals, with an increased level of dependency on family impacting the individual’s sense of self. These findings resonate with findings from other South African studies^3,4,5,6,7,8,18,19,20^ and highlight the challenges that CVA survivors experience across the country regardless of the differences in their geographical location.

This study has highlighted the factors that limit CVA survivors’ participation in daily activities and their community engagement. These factors included many CVA survivors being reluctant to leave their house because of fears of falling or being vulnerable to crime. This resulted in participants remaining in their homes unless there was someone to assist them, which further exacerbated the sense of social isolation and exclusion. A number of other studies have documented the prevalence of fears of falling among CVA survivors.^23,24,25,26,27,28,29,30,31^ In addition to this study, there is only one other study^23^ that highlighted participants’ fears of being mugged or attacked, which may be because of the high rate of crime in SA.

As participants began to feel more like themselves again, they were able to resume previous roles and participation in meaningful activities, such as work or chores in the home, which resulted in an increased sense of self as well as an incentive to participate in rehabilitation because of the observed gains. With an increase in the uptake of their premorbid roles, individuals felt content and proud of their accomplishments and experienced a change of attitude as they started to accept their situations. Study findings from Gretchel et al.,^4^ Wasserman^8^ and Mduzi et al.^5^ corresponded with the authors’ argument that resumption of roles and participation in meaningful activities enabled more successful community reintegration.

Early rehabilitation following CVA is necessary to improve functional outcomes and community reintegration.^8,10,13^ The participants in this study experienced improvements in residual deficits because of rehabilitation, which aided their participation in their occupations. However, a number of the participants reported that they had returned to their home environment without having the benefit of in-hospital rehabilitation and had only started rehabilitation in the community. Wasserman et al.,^8^ Mamabolo et al.^19^ and Kloppers et al.^13^ emphasise the need for inpatient rehabilitation and agree that there are less favourable functional outcomes if the CVA survivor has a short stay in hospital. The current experiences of CVA survivors is contrary to the guidelines proposed in the *Framework and Strategy for Disability and Rehabilitation Services*, which recommends that rehabilitation services be offered at tertiary, district and PHC level.^9^ The lack of inpatient rehabilitation resulted in CVA survivors being discharged with a lack of knowledge of how to cope with their impairments and this possibility contributed towards the lack of referrals between the hospital and the relevant CHC, which meant that the participants were unable to receive continuity of care. Some individuals received outpatient rehabilitation more than 6 months following their CVA, if at all. According to the norms and standards for community-based clinic services within the PHC framework, districts are required to offer CBR and have an effective and efficient referral system between hospital and clinics. Whilst there are services available within the KwaDabeka community, CVA survivors are often missed because of the poor communication channels between the levels of care.

Primary health care facilities are required to provide home-based services and rehabilitation that is comprehensive, holistic and client centred.^14^ However, some participants were unable to access the CHC as a result of environmental factors such as lack of transport or difficulty mobilising in the community to access public transport. They were unable to access community rehabilitation, as the therapists did not offer home visits within the community. Those participants who were able to afford travel expenses, equipment and finance to make adaptations showed improvement in their participation, thereby reducing social isolation and improving participation.^32,33,34^

There is a need to approach rehabilitation from the perspective of CVA survivors and their families, and this approach should draw on the knowledge of the community and the resources available. The participants in this study voiced specific skills and activities that held meaning to them and highlighted the enablers in the community, such as support of their friends. Resources such as families and friends being willing to build ramps or advocating with local councillors or the public works department to promote more universal access in public spaces would help facilitate community reintegration. Wasserman^8^ suggests that any model of community-based stroke care should include the CVA survivor and their caregiver and promote the integration of a CHW into their intervention plan. Despite KwaDabeka having CHWs, none of the participants mentioned receiving assistance from the CHW. The authors would suggest that this is a gap in the service; as the limited number of rehabilitation therapists could not provide adequate service to all CVA survivors, the CHWs must be included in the planning and execution of therapy to enable continuity of care.

Environmental factors such as limited space within the house, steps in the home and community, and steep hills and roads promoted the belief that CVA survivors could not leave their house, which increased their social isolation as this restricted their ability to participate in meaningful activities or socialise with the community or friends.^23,34,35^ Furthermore, the increased distance between the CVA survivors’ homes and rehabilitation services made the service less accessible and more expensive in terms of transport costs. Wheelchair-bound individuals, because of decreased functional mobility, were found to be more isolated and homebound, which heightened their feelings of being socially excluded.^23,34,35^ Social support received by the CVA survivors was seen as an enabler to community reintegration. The support that the individuals received increased their motivation, evoked positive emotions and decreased isolation, which promoted good functional outcomes, role resumption and improved participation in occupations.^34,35,36^ Inevitably this was said to decrease social isolation and led to feelings of being valued, which is essential towards effective community reintegration.^37^

A noteworthy limitation of this study may have been the exclusion of other stakeholders that could have contributed to the holistic understanding of community reintegration of CVA survivors. Successful reintegration may have been more rigorously determined by having the experiences of the CVA survivors triangulated with feedback from their caregivers and possibly the team involved in the community rehabilitation, including the inputs from the CHWs. Moreover, a deeper exploration into the vocational trajectories of some of the CVA survivors who returned to work may have been beneficial in understanding the impact that this may have had on the social experiences of the CVA survivors.

## Conclusion

The quality of life of an individual is impacted following a CVA, with subsequent residual impairments causing activity limitations that hinder successful community reintegration. The factors that limit participation and enablers to community reintegration of CVA survivors, as identified in this study, are in keeping with other South African studies.^3,4,5,6,7,8,9,10^ The findings suggest that, for successful community reintegration, the focus of inpatient and CBR needs to be geared towards specific interventions for CVA survivors that facilitate community reintegration or assisting caregivers to identity potential changes that need to be made to the CVA survivor’s home to create facilitators that promote community reintegration. These include having assistive devices that promote activity participation such as wheelchairs, improving participation in meaningful activities such as leisure, work and domestic-related tasks, and caregiver training to promote carry-over of gain from the inpatient stage into the home. A number of recommendations have surfaced from this study. These include (1) education of CVA survivors on awareness and access to rehabilitation services as well as advice on how to improve CVA survivors’ ability to mobilise in the community and make environmental adaptations to facilitate universal access; (2) provision of home programmes and caregiver training for continuity of care in the home environment; (3) the need for inpatient and community rehabilitation that focuses on fostering and encouraging early independence and participation in ADL; (4) the creation and monitoring of support groups (by the CHCs) for the CVA survivors within different communities (PHC clinics on dates that coincide with their medication collection) to reduce isolation and enhance social support; and finally (5) the inclusion of home-based rehabilitation into current models of care.

## References

[1] Maleka M, Stewart AS, Hale L The experience of living with stroke in low urban and rural socioeconomic areas of South Africa. S Afr J Physiother. 2012;68(3):25–29. 10.4102/sajp.v68i3.21

[2] Pillay-van Wyk V, Msemburi W, Laubscher R, et al. Second national burden of disease study South Africa: National and subnational mortality trends, 1997–2009. Lancet. 2013;381(Suppl 0):S113 10.1016/S0140-6736(13)61367-7

[3] Cunningham N, Rhoda A Outcomes of stroke patients discharged from an in-patient facility in the Eastern Cape, South Africa: A mixed methods design. S Afr J Physiother. 2014;70(3):26–31. 10.4102/sajp.v70i3.265

[4] Gretschel D, Visagie S, Inglis G Community integration of adults with disabilities post discharge from an in-patient rehabilitation unit in the Western Cape. S Afr J Physiother. 2017;73(1):a361 10.4102/sajp.v73i1.361 PMC6093139 30135906

[5] Mudzi W, Stewart A, Musenge E Community participation of patients 12 months post-stroke in Johannesburg, South Africa. Afr J Prim Health Care Fam Med. 2013;5(1), Art. #426:1–9. 10.4102/phcfm.v5i1.426

[6] Rhoda A Limitations in activity and participation experienced by stroke patients: A qualitative inquiry. S Afr J Physiother. 2012;68(3):20–24. 10.4102/sajp.v68i3.20

[7] Rouillard S, De Weerdt W, De Wit L, Jelsma J Functioning at 6 months post stroke following discharge from inpatient rehabilitation. S Afr Med J. 2012;102(6):545–548. 10.7196/SAMJ.5488 22668960

[8] Wasserman S, De Villiers L, Bryer A Community-based care of stroke patients in a rural African setting. S Afr Med J. 2009;99(8):579–583. 19908616

[9] National Department of Health Framework and strategy for disability and rehabilitation services [homepage on the Internet]. 2015 [cited 2018 Nov 1]. Available from: http://ilifalabantwana.co.za/wp-content/uploads/2016/07/Framework-25-may_1_3.docx

[10] Hassan S, Visagie S, Mji G The achievement of community integration and productive activity outcomes by CVA survivors in the Western Cape Metro Health District. S Afr J Occup Ther. 2012;42(1):11–16.

[11] Ned L, Cloete L, Mji G The experiences and challenges faced by rehabilitation community service therapists within the South African Primary Healthcare health system. Afr J Disabil. 2017;6:1 10.4102/ajod.v6i0.311 PMC5645568 29062760

[12] Sherry K Essential considerations for equitable, accessible and poverty-reducing health care in South Africa. S Afr Health Rev. 2014;15:89.

[13] Kloppers M, Pretorius B, Vlok ED Clients’ subjective experience of their participation in rehabilitation at an out-patient community rehabilitation center. S Afr J Occup Ther. 2016;46(1):59–63. 10.17159/2310-3833/2016/v46n1a12

[14] Rispel L Analysing the progress and fault lines of health sector transformation in South Africa. S Afr Health Rev. 2016;2016(1):17–23.

[15] McColl MA, Shortt S, Godwin M, et al Models for integrating rehabilitation and primary care: A scoping study. Arch Phys Med Rehabil. 2009;90(9):1523–1531. 10.1016/j.apmr.2009.03.017 19735780

[16] Dijkers M Community integration: Conceptual issues and measurement approaches in rehabilitation research. Top Spinal Cord Inj Rehabil. 1998;4(1):1–5. 10.1310/BJJA-2018-45KL-0VTL

[17] Mothabeng DJ, Eksteen CA, Westaway M Psychometric validation of the reintegration to normal living index in people living with spinal cord injuries. S Afr J Physiother. 2012;68(2):29–32. 10.4102/sajp.v68i2.13

[18] Cawood J, Visagie S, Mji G Impact of post-stroke impairments on activities and participation as experienced by stroke survivors in a Western Cape setting. S Afr J Occup Ther. 2016;46(2):10–15. 10.17159/2310-3833/2016/v46n2a3

[19] Mamabolo MV, Mudzi W, Stewart AS, Olorunju S, Singh A A study to determine post discharge functional improvements in patients with stroke. S Afr J Occup Ther. 2009;39(1):15–18.

[20] Pang MYC, Eng JJ, Miller WC Determinants of satisfaction with community reintegration in older adults with chronic stroke: Role of balance self-efficacy. Phys Ther. 2007;87(3):282–291. 10.2522/ptj.20060142 17284545

[21] Creswell JW, Poth CN Qualitative inquiry and research design: Choosing among five approaches 4th ed. Thousand Oaks, CA: Sage; 2017.

[22] Saldaña J. The coding manual for qualitative researchers 2nd ed. Thousand Oaks, CA: Sage; 2015 1–303.

[23] Cawood J, Visagie S Environmental factors influencing participation of stroke survivors in a Western Cape setting. Afr J Disabil. 2015;4(1):1–9. 10.4102/ajod.v4i1.198 PMC5433486 28730037

[24] Chimatiro GL Barriers to reintegration experienced by stroke clients post discharge from a rehabilitation center in Malawi [homepage on the Internet]. 2012 [cited 2018 Nov 1]. Available from: https://etd.uwc.ac.za/bitstream/handle/11394/4344/Chimatiro_msc_2012.pdf?sequence=1&isAllowed=y

[25] Dewey H, Sherry L, Collier J Stroke rehabilitation 2007: What should it be? Int J Stroke. 2007;2(3):191–200. 10.1111/j.1747-4949.2007.00146.x 18705943

[26] Manaf H, Justine M, Omar M, Isa KA, Salleh Z Turning ability in stroke survivors : A review of literature. ISRN Rehabil. 2012;2012:284924 10.5402/2012/284924

[27] Dowswell G, Lawler J, Dowswell T, Young J, Forster A, Hearn J Investigating recovery from stroke: A qualitative study. J Clin Nurs. 2000;9(4):507–515. 10.1046/j.1365-2702.2000.00411.x 11261130

[28] Andersson G, Kamwendo K, Appelros P Fear of falling in stroke patients: Relationship with previous falls and functional characteristics. Int J Rehabil Res. 2008;31(3):261–264. 10.1097/MRR.0b013e3282fba390 18708851

[29] Schmid AA, Rittman M Fear of falling: An emerging issue after stroke. Top Stroke Rehabil. 2007;14(5):46–55. 10.1310/tsr1405-46 17901015

[30] Rosén E, Sunnerhagen KS, Kreuter M Fear of falling, balance, and gait velocity in patients with stroke. Physiother Theor Pract. 2005;21(2):113–120. 10.1080/09593980590922299 16392464

[31] Lee KB, Lim SH, Kim KH, et al Six-month functional recovery of stroke patients: A multi-time-point study. Int J Rehabil Res. 2015;38(2):173 10.1097/MRR.0000000000000108 25603539 PMC4415968

[32] Gustafsson L, Bootle K Client and carer experience of transition home from inpatient stroke rehabilitation. Disabil Rehabil. 2013;35(16):1380–1386. 10.1097/MRR.0000000000000108 23244213

[33] Schulz CH, Hersch GI, Foust JL, et al Identifying occupational performance barriers of stroke survivors: Utilization of a home assessment. Phys Occup Ther Geriatr. 2012;30(2):109–123. 10.3109/02703181.2012.687441 PMC3839531 24285912

[34] Jellema S, Van Hees S, Zajec J, Van der Sande R, Nijhuis-van der Sanden MW, Steultjens EM What environmental factors influence resumption of valued activities post stroke: A systematic review of qualitative and quantitative findings. Clin Rehabil. 2017;31(7):936–947. 10.1177/0269215516671013 27681480 PMC5482381

[35] Chimatiro G, Rhoda A Environmental barriers to reintegration experienced by stroke clients post discharge from a rehabilitation centre in Malawi. S Afr J Physiother. 2014;70(1):18–23. 10.4102/sajp.v70i1.260

[36] Dobkin B Rehabilitation after stroke. New Engl J Med. 2005;352(16):1677–1684. 10.1056/NEJMcp043511 15843670 PMC4106469

[37] Kahonde CK, Mlenzana N, Rhoda A Persons with physical disabilities’ experiences of rehabilitation services at community health centres in Cape Town. S Afr J Physiother. 2010;66(3):2–7. 10.4102/sajp.v66i3.67

[38] National Department of Health The primary health care package for SA – A set of norms and standards [homepage on the Internet]. 2003 [cited 2018 Nov 1]. Available from: https://www.westerncape.gov.za/general-publication/primary-health-care-package-sa-set-norms-and-standards

[39] Venter C, Bogopane H, Rickert T Enhanced accessibility for people with disabilities living in urban areas [serial online]. 2002 [cited 2018, March 30] Available from: http://digitalcommons.ilr.cornell.edu/gladnetcollect/257/

[40] Linder SM, Rosenfeldt AB, Reiss A, et al The home stroke rehabilitation and monitoring system trial: A randomized controlled trial. Int J Stroke. 2013;8(1):46–53. 10.1111/j.1747-4949.2012.00971.x 23280269 PMC4362540

